# A Treatise on Diseases of the Bones

**Published:** 1850-01

**Authors:** 


*A treatise on diseases of the Bones.*—By Edward Stanley, F. R. S. President of the Royal College of Surgeons of England, and Surgeon to St. Bartholomew's Hospital. Philadelphia, Lea and Blanchard, 1849, 12 mo. pp. 286.

The following quotation from the preface will enable the reader to appreciate the character of this work.

"Observations, continued with some diligence through many years, and, in a large field of experience, has enabled me to accumulate the materials upon which the present volume is founded. The size of the volume bears no proportion to the number of the facts out of which it is constructed. For in this, as in other scientific investigations, the first object has been to obtain the facts, and the sedond to interpret them rightly, for the conclusions they warrent. This I have endeavoured to do, but with the result, I am aware, of leaving some of the morbid phenomena of bone uninterrupted, even unnoticed, for the reason that they were to me unintelligible. And I am induced to submit the work in its incomplete state, rather than detail in it characters of diseases from which, in consequence of not understanding them, I should not be able to deduce either pathological conclusions or indications of treatment.

Sold by G. H. Derby & Co.

